# Mitochondrial Dysfunction: An Emerging Link in the Pathophysiology of Cardiorenal Syndrome

**DOI:** 10.3389/fcvm.2022.837270

**Published:** 2022-02-25

**Authors:** Shuqing Shi, Bingxuan Zhang, Yumeng Li, Xia Xu, Jiayu Lv, Qiulei Jia, Ruoning Chai, Wenjing Xue, Yuan Li, Yajiao Wang, Huaqin Wu, Qingqiao Song, Yuanhui Hu

**Affiliations:** ^1^Department of Internal Medicine, Guang'anmen Hospital, China Academy of Chinese Medical Sciences, Beijing, China; ^2^Beijing University of Chinese Medicine, Beijing, China; ^3^Department of Cardiovascular, Guang'anmen Hospital, China Academy of Chinese Medical Sciences, Beijing, China; ^4^Reproductive and Genetic Center, Affiliated Hospital of Shandong University of Traditional Chinese Medicine, Jinan, China

**Keywords:** cardiorenal syndrome, mitochondrial dysfunction, heart failure, kidney failure, oxidative stress, inflammation

## Abstract

The crosstalk between the heart and kidney is carried out through various bidirectional pathways. Cardiorenal syndrome (CRS) is a pathological condition in which acute or chronic dysfunction in the heart or kidneys induces acute or chronic dysfunction of the other organ. Complex hemodynamic factors and biochemical and hormonal pathways contribute to the development of CRS. In addition to playing a critical role in generating metabolic energy in eukaryotic cells and serving as signaling hubs during several vital processes, mitochondria rapidly sense and respond to a wide range of stress stimuli in the external environment. Impaired adaptive responses ultimately lead to mitochondrial dysfunction, inducing cell death and tissue damage. Subsequently, these changes result in organ failure and trigger a vicious cycle. *In vitro* and animal studies have identified an important role of mitochondrial dysfunction in heart failure (HF) and chronic kidney disease (CKD). Maintaining mitochondrial homeostasis may be a promising therapeutic strategy to interrupt the vicious cycle between HF and acute kidney injury (AKI)/CKD. In this review, we hypothesize that mitochondrial dysfunction may also play a central role in the development and progression of CRS. We first focus on the role of mitochondrial dysfunction in the pathophysiology of HF and AKI/CKD, then discuss the current research evidence supporting that mitochondrial dysfunction is involved in various types of CRS.

## Introduction

The crosstalk between the heart and kidney plays an important role in regulating fluid balance, metabolite excretion, and neuroendocrine function to maintain homeostasis (1), and there are common pathological risk factors between these two organs. Cardiorenal syndrome (CRS) is broadly defined as “a disorder of the heart and kidney whereby acute or chronic dysfunction in one organ may induce acute or chronic dysfunction of the other” (2, 3). In 2010, the Acute Dialysis Quality Initiative (ADQI) classified CRS into five types based on the organ leading to the syndrome and the time course of disease progression (i.e., acute or chronic) (2). Epidemiological studies have shown that 25–63% of patients with heart failure (HF) suffered from a form of CRS defined by the ADQI (4). According to the Global Burden of Disease Study, the global prevalence of chronic kidney disease (CKD) in 2015 was estimated to be approximately 323 million, and 10–47% of CKD patients had cardiovascular disease, which was the leading cause of death in the CKD population (5). Furthermore, as renal function declined, the risk of all-cause mortality increased from 20% in stage 3a to nearly 500% in stage 5. Moreover, 25–50% of patients with HF had CKD, including an estimated 5.7 million people in the United States. Mortality increased by 56% in HF patients with concomitant CKD and further increased to 131% in patients with moderate or severe renal impairment (6, 7).

**Figure 1:**
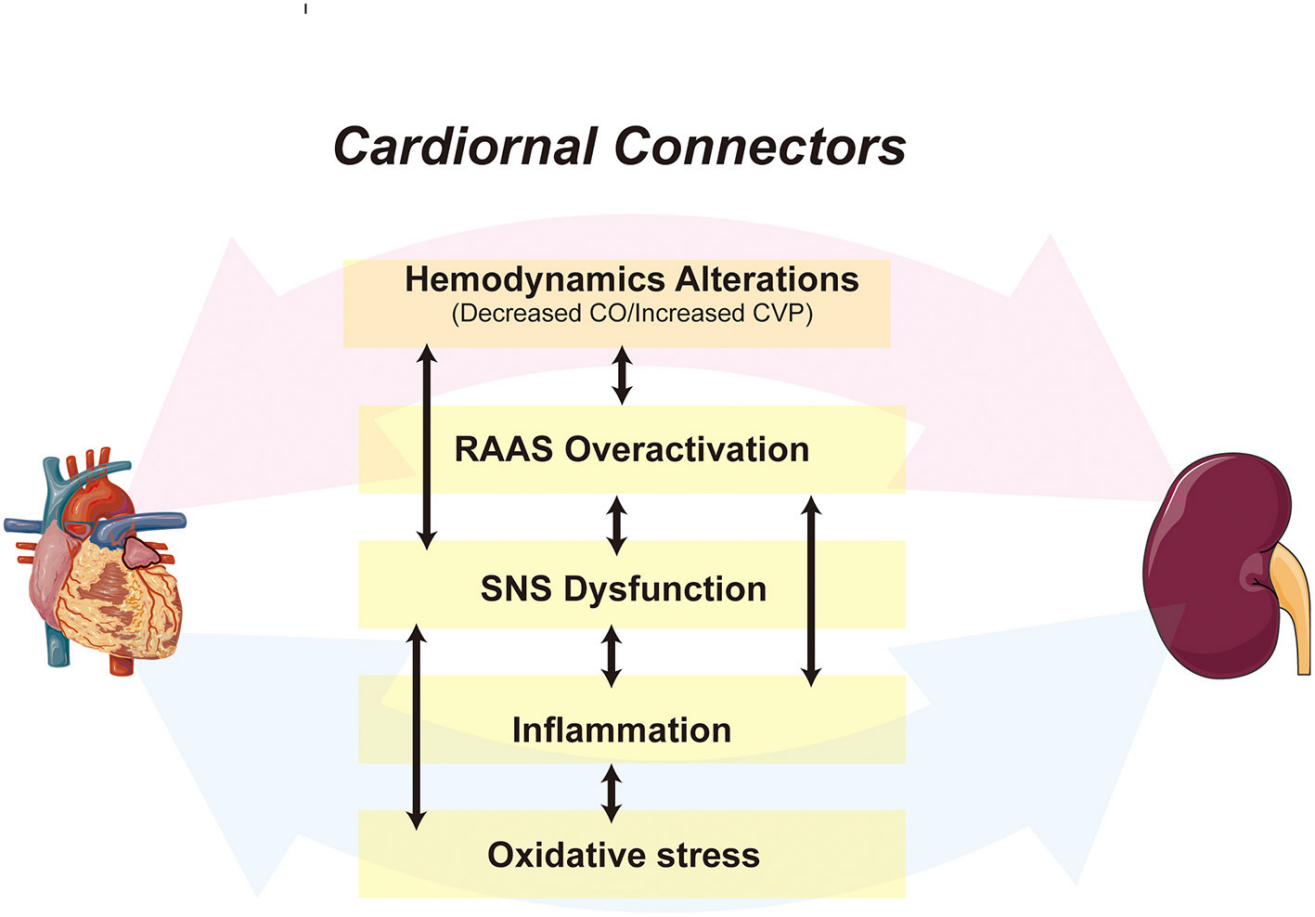
A schematic depiction of the cardiorenal connectors in CRS. The cardiorenal connectors, including hemodynamic factors, RAAS, SNS, inflammation, and oxidative stress are core mechanisms of CRS, all of which synergize and activate each other, leading to further deterioration of cardiac and renal function. CO, Cardiac Output; CVP, central venous pressure.

Despite decades of basic and clinical research, CRS remains a significant public health burden due to its unclear pathogenesis and lack of effective treatment (8). Both in acute and chronic settings, the pathophysiological pathways that exacerbate the cardiac or renal injury, such as persistent activation of the renin-angiotensin-aldosterone system (RAAS) and sympathetic nervous system (SNS), chronic inflammation, oxidative stress, and fibrosis, play a vital role in CRS (9, 10) (Figure 1). Although the above theoretical mechanisms explain the pathological changes in CRS, there is no single-factor hypothesis to describe how damage to an organ triggers distal organ dysfunction or failure.

It is widely known that the heart contains the highest amounts of mitochondria in the human body. Given the high energy consumption of cardiomyocytes, more than 90% of the ATP is produced by mitochondria, which account for approximately one-third of the cardiomyocyte volume (11). At the renal level, owing to the high energy demands for solute reabsorption (12), the kidney, especially the cells in proximal tubules and medullary thick ascending limb, contains abundant mitochondria (13). A wide range of stress stimuli, such as ischemia, hypoxia or toxic injury, primarily target the cardiomyocytes as well as tubular epithelial cells especially the highly metabolically active proximal tubular segment (14, 15). In fact, mitochondria rapidly sense and respond to the insults to maintain their homeostasis through morphological alterations, bioenergetics adaptations, and enhanced self-renewal/degradation (12, 16). Impaired adaptive responses are closely associated with a decline in cardiac/renal function through various mechanisms affecting metabolism, oxidative stress, inflammation, calcium dynamics and mitophagy, and it is widely recognized that mitochondrial malfunction is an early and prominent signature of organic depression. Therefore, disrupted mitochondrial homeostasis is considered not only as a consequence of myocardial injury but also as a possible cause of HF (17). Meanwhile, Plotnikov and colleagues concluded that mitochondrial dysfunction was an independent risk factor for CKD regardless of the underlying etiology (18), and it was closely associated with the progression of CKD (19). Maintaining mitochondrial function significantly preserves the structural integrity of the left ventricular myocardium and renal parenchyma and the function of the heart and kidney (20, 21).

**Figure 2:**
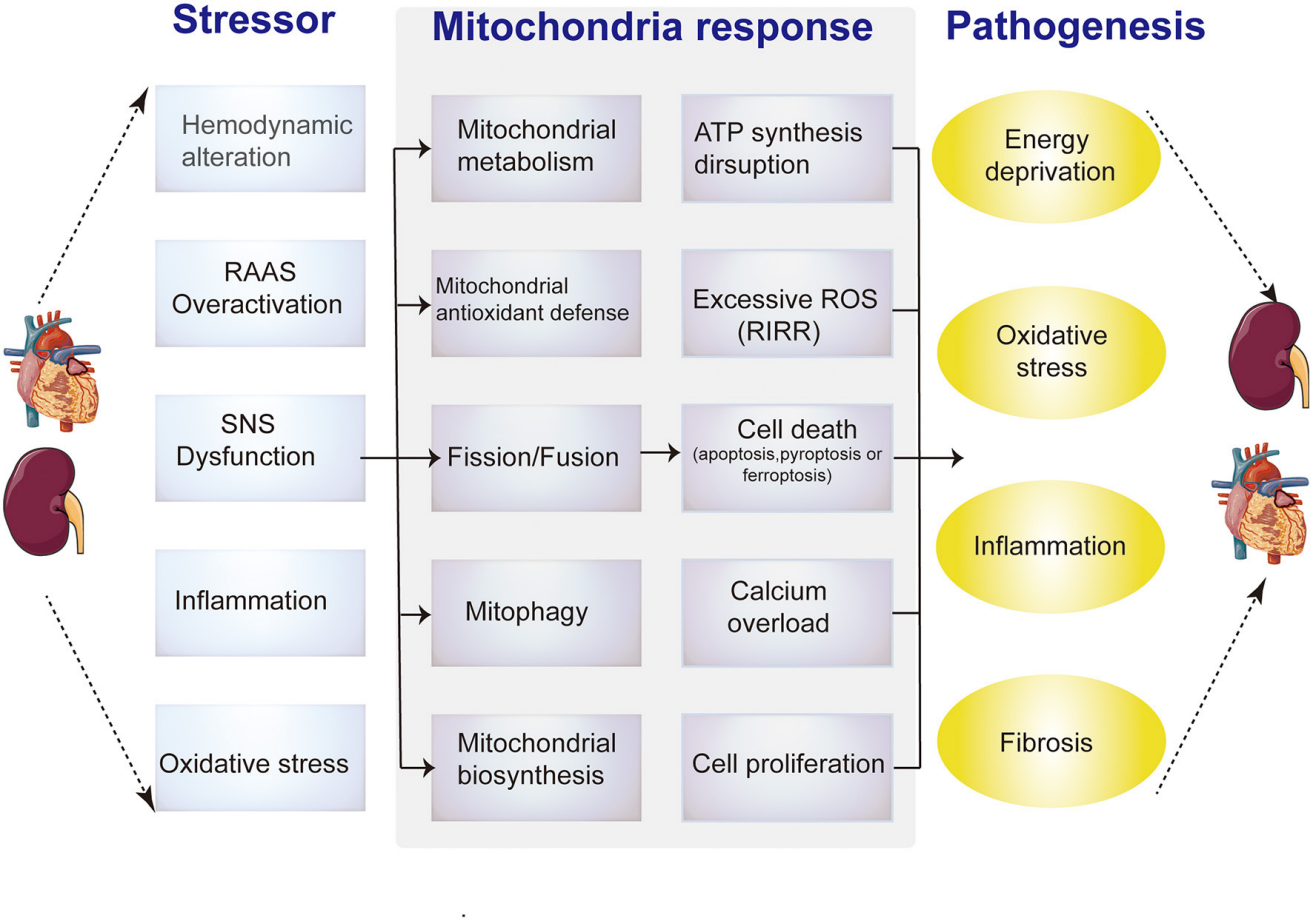
Diagrammatic representation shows the relationship between pathological alterations in CRS and mitochondrial dysfunction mechanisms. Intrinsic and extrinsic stress signals, such as hemodynamic alteration, RAAS overactivation, SNS dysfunction, inflammation or oxidative stress, can activate mitochondrial responses, including mitochondrial metabolism, antioxidant defense imbalance, abnormal dynamics, mitophagy and biogenesis. Impaired adaptive responses ultimately lead to mitochondrial dysfunction, include ATP synthesis reduction, excessive ROS, aberrant calcium signaling, (de) differentiation or cell death (apoptosis or necrosis). These changes in cardiomyocytes or renal tubular epithelial cells induce energy deprivation, oxidative stress, inflammation and fibrosis.

Furthermore, all the above-mentioned pathological factors in CRS damage the mitochondria in distal organs through circulatory effects (21–23), suggesting that mitochondria play a connecting role in cardiorenal interaction, and may be a core link in CRS progression (Figure 2). However, the specific mechanism and evidence have not been well summarized. In this article, we first outline the unique characteristics of heart and kidney mitochondria and their important role in organ injury, then summarize the current evidence of mitochondrial involvement in the pathogenesis of various types of CRS, including the potential of mitochondria-targeted CRS therapy.

## Impaired Mitochondrial Metabolism

**Figure 3:**
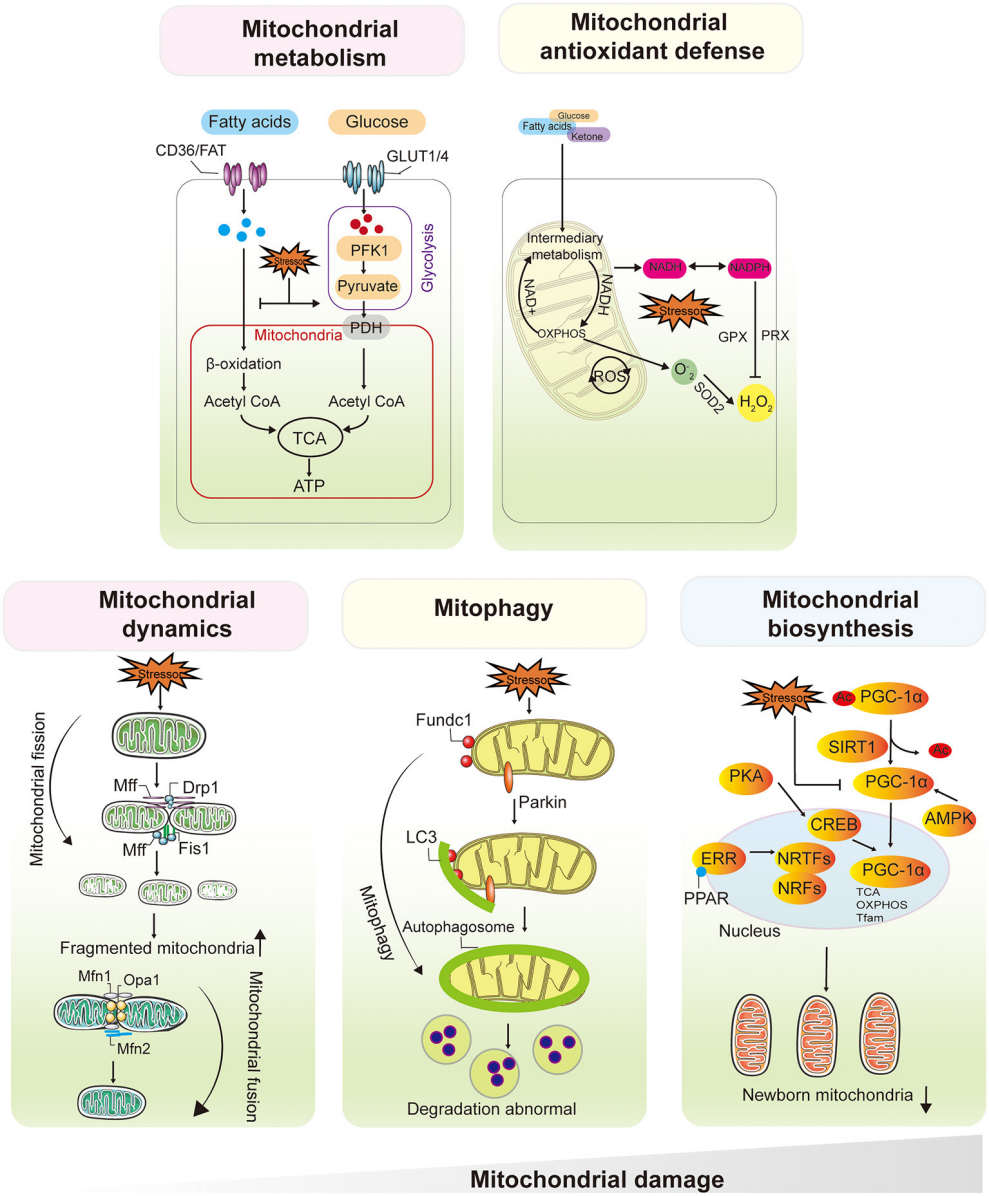
Mitochondrial Dysfunction Plays a Central and Multifaceted Role in HF and AKI/CKD. Mitochondria from cardiomyocytes and renal proximal tubular cells preferentially use fatty acyl-CoA, the primary substrate for mitochondrial FAO, rather than pyruvate to generate ATP. The hallmarks of metabolic remodeling are FAO downregulation and increased glucose utilization, which are observed in both early-stage heart and kidney injury. ROS were initially thought to be by-products of mitochondrial OXPHOS, the balance between mtROS production and scavenging is critical for maintaining mitochondrial function and cell viability. Mitochondrial dynamics contribute to the functional integrity of mitochondria. Fusion allows mixing of contents within the mitochondrial network and protects the mitochondria from stress. Damaged mitochondria undergo selective mitochondrial autophagy *via* Parkin, Fundc1, through autophagosome formation and lysosome-mediated degradation. Upregulation of PGC1α and activation of NRF1/2 initiate mitochondrial biogenesis, followed by mtDNA amplification and synthesis of nuclear-encoded mitochondrial proteins. After severe mitochondrial damage, increased fission leads to mitochondrial fragmentation, depolarization of the mitochondrial membrane potential inhibits fusion, and frequent abnormal fission events will affect mitochondrial autophagy, leading to abnormal degradation of damaged mitochondria, along with a decrease in the number and quality of nascent mitochondria mediated by mitochondrial biosynthesis. NRFs, nuclear respiratory factors, PGC1α, PPARγ coactivator-1α. FAO, fatty acid β-oxidation, OXPHOS, oxidative phosphorylation.

Under physiological conditions, mitochondria from adult cardiomyocytes and renal proximal tubular cells (PTCs) preferentially use fatty acyl-CoA, the primary substrate for mitochondrial fatty acid β-oxidation (FAO), rather than pyruvate to generate ATP (24, 25). It has been estimated that fatty acids supply ~60–90% of the energy used to synthesize ATP in cardiomyocytes, whereas 10–40% of ATP is derived from glucose metabolism (26). PTCs almost exclusively depend on FAO and subsequent mitochondrial oxidative phosphorylation (OXPHOS) as their sole energy source (25). When faced with various biological stresses, such as temporary hypoxia, FAO in most hypermetabolic cells shuts down for a period of time, allowing the flexible conversion of metabolic substrates from FAs to glucose (i.e., the Randle cycle, proposed in 1963) (27, 28). This switch compensates for impaired energy in a short period and exerts a partial organ protective effect. However, the conversion affects the activity of electron transport chain (ETC)-related proteins, leading to impaired OXPHOS and restricted ATP production (29, 30). The hallmarks of metabolic remodeling are FAO downregulation and increased glucose utilization, which are observed in both early-stage heart and kidney injury (31, 32) (Figure 3).

### Role in Cardiac Injury

As a central mechanism of energy deprivation in the failing heart, impaired mitochondrial metabolism has inspired decades of research to date. *In vitro* studies have shown that ATP is reduced in failing cardiomyocytes, particularly in mitochondria (33, 34). Both animal models and clinical trials have demonstrated that ATP flux is reduced in advanced HF, and ATP supplementation improves cardiac function and even reverses the subsequent structural remodeling of ventricles (34–36). The utilization of myocardial FAs, which are metabolized *via* non-oxidative pathways to produce lipotoxic ceramides and diacylglycerols in rats with severe HF, leading to mitochondrial dysfunction and apoptosis and further aggravating the impaired energy metabolism (37). Previous studies confirmed that a greater dependence on glucose in the failing heart promotes glycolysis, leading to increased lactate production and anaplerosis (38). It also drives more glucose into collateral pathways, all of which reduce the efficiency of ATP synthesis and exacerbate pathological myocardial remodeling (38, 39).

### Role in Kidney Injury

Experimental evidence indicates that FAO dysregulation profoundly affects the fate of PTCs by promoting inflammation, ultimately leading to mesenchymal fibrosis and epithelial-to-mesenchymal transition (40). High levels of albumin-bound long-chain saturated FAs promote the progression of renal tubular damage and interstitial fibrosis through the activation of pro-inflammatory pathways, including tumor necrosis factor-alpha (TNF-α), CC motif chemokine 2, and interleukin (IL)-6, and increase the production of ROS (40–42). Kang HM et al. reported that PTCs treated with the CPT-1 inhibitor etomoxir undergo morphologic and genomic changes, with increased expression of mesenchymal genes, such as *ACTA2* (encoding α-smooth muscle actin), *VIM* (encoding vimentin), and *COL1A1* and *COL3A1* (encoding fibrillar collagens) (43). In addition, Lan R et al. found that the shift from FAO to glycolysis in PTCs undergoing renal ischemia/reperfusion (I/R) injury promoted the development of tubular atrophy and transition from AKI to CKD (44). This may be related to the increased expression of the key glycolytic enzyme glycolysis pyruvate kinase M2 and levels of lactate metabolites, cooperatively leading to hypoxic and acidic environments and eventually inhibiting the proliferation and differentiation of podocytes and aggravating fibrosis (45, 46).

## Mitochondrial Antioxidant Defense Imbalance

ROS were initially thought to be by-products of mitochondrial OXPHOS. Electrons that leak out of the major site of complex I and complex III in the ETC and react with O_2_ generate superoxide anions (47), which are then converted to H_2_O_2_ by superoxide dismutase (48). Mitochondrial antioxidant systems, including catalase, glutathione peroxidases, and peroxiredoxin, further reduce H_2_O_2_ to water. Under physiological conditions, the mitochondrial antioxidant defense system maintains low levels of ROS in the organelle (49, 50). Under stress conditions, ROS accumulate when mitochondrial ROS (mtROS) production exceeds the mitochondrial antioxidant defense capacity or when the antioxidant defense system is impaired (51). Subsequently, mtROS react rapidly with nitric oxide to form a potent oxidant and nitrifying agent. As a result of mtROS accumulation, H_2_O_2_ release from mitochondria to the cytoplasm increases, exacerbating oxidative damage outside the mitochondria (52, 53). Therefore, the balance between mtROS production and scavenging is critical for maintaining mitochondrial function and cell viability (Figure 3).

### Role in Cardiac Injury

In the failing heart, damage caused by excessive mtROS and innate antioxidant defense exhaustion are evident in human patients and animal models (54–56). The overproduction of mtROS is primarily due to increased expression and activity of NADPH oxidase through a variety of pathological stimuli, including mechanical stretch, angiotensin II, endothelin 1, and TNF-α (52). Moreover, experimental evidence suggests the activities of superoxide dismutase, catalase, and glutathione peroxidases are significantly reduced during HF (17). Mitochondria-targeted ROS scavenging (e.g., MitoQ, MitoTEMPO, or SS-31) has demonstrated benefit in animal models of HF (57–59). Interestingly, NAD^+^, a precursor for the phosphorylated dinucleotide pair NADP^+^/NADPH (plays a major role in the detoxification of ROS), was also significantly reduced in the myocardium of some HF mice (60). In addition, nicotinamide mononucleotide adenosyltransferase, an enzyme responsible for NAD^+^ production, was found to be substantially inhibited in both mouse HF models and patients (61, 62). Further studies indicate that increasing intracellular NAD^+^ levels by pharmacological or genetic approaches restore protein acetylation and improve cardiac function in mouse models of HF (63).

### Role in Kidney Injury

Experimental evidence suggests that kidney injury is accompanied by an impaired mitochondrial antioxidant defense system. Increased production of mtROS is a common feature of AKI and CKD (64, 65). Enhanced mitochondrial antioxidant defenses by supplementation with mitochondria-targeted antioxidants have been shown to attenuate mitochondrial dysfunction and reduce kidney injury in animal models of IR-induced renal fibrosis, diabetic nephropathy, and unilateral ureteral obstruction (UUO)-induced CKD (66–68). Notably, a moderate increase in mtROS may regulate signaling pathways involved in renal injury and incomplete renal repair (69). For example, mtROS have been shown to activate hypoxia-inducible factor 1α in response to hypoxia; the NLRP3 pathway, which induces inflammation, cytokine production, and innate immune responses; and the transforming growth factor-β (TGFβ) pathway, which has a pro-fibrotic effect in disease conditions (70–72).

Noteworthily, excessive mtROS have been shown to affect a wide range of cellular functions in the context of HF and AKI/CKD. Mitochondrial damage caused by an initial increase in oxidative stress leads to a further increase in ROS production and more severe damage. This results in so-called ROS-induced ROS release in mitochondria, creating a vicious cycle and increasing the tendency for cell death (73). High levels of ROS damage mtDNA and induce mtDNA leakage, which can bind to Toll-like receptors or nucleotide-binding oligomerization domain-containing protein (NOD)-like receptors, leading to inflammation (74). Moreover, increased ROS also induce mitochondrial permeability transition pore opening, lead to cell death, and impair mitochondrial biogenesis (25, 75, 76). However, the specific role of these mechanisms in the development of damage remains to be fully elucidated.

## Abnormal Mitochondrial Dynamics

Mitochondria are not static organelles. In fact, they continuously undergo fusion and fission, two processes that define mitochondrial dynamics (77). Through fission and fusion, mitochondrial size, distribution, shape, position, and mass are fine-tuned in response to changes in the metabolic state of the cell (77). Mitochondrial fission is a multistep process that allows a mitochondrion to split in two daughter mitochondria, and primarily mediated by dynamin-related protein 1 (Drp1), a large dynamin-related guanosine triphosphate hydrolase (GTPase) (78, 79). During the process, Drp1 is recruited to the outer mitochondrial membrane, and then GTP hydrolysis enhances membrane contraction, leading to the recruitment of dynamin 2 to terminate membrane cut-off (80). In contrast, mitochondrial fusion is driven by a two-step process induced by several GTPases, including mitofusin 1 (Mfn1) and Mfn2 involved in outer mitochondrial membrane fusion and optic atrophy 1 (Opa1) involved in inner membrane fusion (81, 82). The continuous alternation between fission and fusion maintains mitochondrial homeostasis and cellular function in response to physiological changes (83). However, the disruption of dynamic balance alters mitochondrial morphology and impairs mitochondrial function, leading to cell viability, which is closely associated with heart and kidney diseases (78, 84) (Figure 3).

### Role in Cardiac Injury

Impaired mitochondrial fission may cause dilated cardiomyopathy, whereas impaired mitochondrial fusion leads to hypertrophy (85–87). Genetic ablation of genes associated with fission/fusion can lead to significant myocardial structural abnormalities, such as enlarged left ventricles, reduced cardiac output, and abnormal myocardial fibrosis (88, 89). Mice with a cardiomyocyte-specific conditional knockout of Drp1 die after 8–13 weeks and develop evidence of HF. At the cellular level, these hearts contain elevated mitochondrial counts, elongated mitochondria, and increased apoptosis (86). Similarly, myocardial Drp1 levels are elevated in HF patients through a mechanism potentially regulated by neurohormone norepinephrine-mediated myocardial hypertrophy. In contrast to mitochondrial fission, the mRNA transcription and protein expression of Mfn1/2 or Opa1 were enhanced in mouse cardiomyocytes (90), suggesting that under physiological conditions, mitochondrial fusion activity in cardiomyocytes is restricted by inhibitory mechanisms. Because of the redundant expression of Mfn1 and Mfn2 in cardiomyocytes, the ablation of either gene fails to significantly inhibit mitochondrial fusion in normal cardiomyocytes (91). At the subcellular level, most mitochondria in Mfn1/Mfn2 double-knockout cardiomyocytes are spherical and lack an elongated morphology, suggesting an imbalance between fission and fusion (92). Interestingly, in Drosophila and mice, Opa1 gene ablation induces mitochondrial fragmentation with mtROS production and respiratory dysfunction, which produces a cardiac phenotype different from that induced by Mfn1/Mfn2 ablation (93). Moreover, heart tube-specific Opa1 knockdown induces dilation of the heart tube, with severe contractile impairment (94). Conversely, Opa1 overexpression *in vivo* refines myocardial injury, and the underlying mechanism may be dependent on the stabilization of the mitochondrial cristae structure of Opa1, which increases mitochondrial respiratory function and prevents cytochrome c release, ROS production, and apoptosis (95).

### Role in Kidney Injury

Mitochondrial fragmentation due to excessive fission and/or fusion inhibition is thought to be a key event in mitochondrial damage and renal tubular injury during AKI (12). In murine models of AKI induced by renal IR or cisplatin toxicity, mitochondrial fragmentation precedes tubular cell apoptosis, and the inhibition of fission attenuates tubular cell death and renal injury (96, 97). Consistent with this finding, proximal tubule-specific deletion of Drp1 protects mice from renal IR-induced tubular cell death, inflammation, and renal injury and accelerates renal recovery (98). *In vitro* studies have shown that Mfn2 deficiency enhances ATP depletion-induced cell injury and death (99). However, proximal tubule-specific Mfn2 knockout mice exhibit milder renal injury and higher survival rates compared with wild-type mice. After AKI in these mice, Mfn2 deficiency stimulates mitogen-activated protein kinase signaling pathway-dependent renal tubular cell proliferation, which may accelerate renal repair and thus overcome the detrimental effects of inhibiting mitochondrial fusion, leading to renal protection (99). Enhanced mitochondrial fragmentation in renal tubular cells and podocytes has been reported in experimental models of diabetic kidney disease (DKD) and renal biopsy samples from patients with DKD (100, 101). In addition, the knockdown of podocyte Drp1 blocked mitochondrial fragmentation, improved mitochondrial fitness, and protected mice from DKD progression (102). Consistent with these findings, pharmacological inhibition of Drp1 protected mice from DKD progression (101, 103). As mentioned above, mitochondrial fragmentation in renal tubular cells may reduce energy metabolism and increase ROS formation, thereby promoting tissue injury, inflammation, and maladaptive renal repair. The specific mechanisms underlying the deleterious role of mitochondrial fragmentation in renal repair need to be investigated in depth.

## Abnormal Mitophagy

Mitophagy is a mechanism that selectively degrades excess and defective mitochondria (104, 105). Two major mechanisms for labeling mitochondria and transporting them to autophagosomes have been identified: one regulated by the serine/threonine-protein kinase PTEN-induced kinase 1 (PINK1), mitochondrial E3 ubiquitin-protein ligase parkin pathway and the other mediated by mitophagy receptors, including BCL-2/adenovirus E1B 19 kDa protein-interacting protein 3 (BNIP3), BCL-2/adenovirus E1B 19 kDa protein-interacting protein 3-like (BNIP3L), FUN14 domain-containing 1 (FUNDC1), and E3 ubiquitin-protein ligase SMURF1 (106, 107). Among them, Parkin and Fundc1 appear to be inducers of protective mitophagy, whereas lethal mitophagy is more likely triggered by the upregulation of Bnip3 and Nix (106, 108). Specifically, although autophagy/mitophagy is considered by most researchers to be the “guardian” of mitochondrial function and cardiomyocyte homeostasis, different adaptors trigger mitophagy to varying degrees, promoting cell survival or cell death. Moderate mitophagy selectively removes impaired mitochondrial subsets, thereby increasing ATP production. In contrast, excessive mitophagy induces cell death by depleting cellular ATP reserves (109). It is worth noting that the degree of mitophagy activation also depends on the level and duration of cellular stress. However, the extent to which mitophagy activation contributes to mitochondrial dysfunction and cellular energy deficiency in HF or AKI/CKD remains unclear (Figure 3).

### Role in Cardiac Injury

Impaired mitophagy in cardiomyocytes has been reported in experimental models of HF and cardiac biopsy samples from patients with HF. In a mouse model of HF induced by transverse aortic coarctation, PINK1 phosphorylation was reduced, accompanied by inhibited mitophagy and impaired mitochondrial function (110). Genetic ablation of Parkin disrupts the mitochondrial network, reduces ATP synthesis, and causes ROS overload in cardiomyocytes (111). AMPKα2 phosphorylates Ser495 of PINK1 to enhance pink/parkin pathway signaling, activate mitophagy, promote the elimination of damaged mitochondria, and improve mitochondrial function, thereby preventing the early progression of HF (110). Interestingly, the role of parkin-mediated mitophagy in the regulation of mitochondrial dynamics has also been reported. In parkin-deficient hearts and cardiomyocytes, mitochondria show fragmentation, which may be related to parkin-mediated inhibition of Mfn1/2 ubiquitination, leading to reduced mitochondrial fusion (110, 112). Furthermore, hearts with baseline genetic ablation of Fundc1 exhibit reduced early to late ventricular filling velocities, prolonged isovolumetric relaxation times, reduced ejection fractions, and decreased fractional shortening, suggesting that Fundc1 knockout mice have impaired cardiac function and are susceptible to HF (113). As a cardiomyocyte death factor, Nix regulates mitochondrial death and acts as a downstream effector of the stress-related hormone norepinephrine, which promotes cardiac fibrosis during ventricular remodeling. In addition, Nix overexpression promotes collagen and fibronectin expression (114).

### Role in Kidney Injury

Increasing evidence indicates that mitophagy plays an essential role in kidney injury. In a mouse model of septic AKI induced by cecum ligation and puncture, renal tubular cells exhibited increased mitophagy in the early stages of septic AKI, followed by decreased mitophagy in the late stages of AKI (115). Increased mitochondrial loss and autophagosome formation have been reported in the regeneration of PTCs after renal IRI, and these abnormalities resolved in normally repaired tubules but persisted and progressively worsened in undifferentiated tubular cells, suggesting a role for mitophagy in renal repair after AKI (44). Furthermore, in the renal tubules of DKD mice, reduced expression of PINK1 and parkin in PTCs, decreased autophagosome formation, and optineurin overexpression enhanced mitophagy, thereby attenuating cellular senescence, mtROS accumulation, and NLRP3 inflammasome activation (116, 117). In addition to DKD, mitophagy has been associated with the development of non-diabetic CKD, with increased mitochondrial PINK1 and parkin formation and increased levels of autophagy in renal tubules and hypoxia-exposed PTCs in UUO mice, suggesting that mitophagy is activated in these settings. In contrast, another study reported reduced parkin expression and autophagy levels in kidney tissue from UUO mice (118, 119). These conflicting findings suggest the presence of context- and cell-type-specific alterations in mitophagy in CKD. However, both studies suggest that the deletion of PINK1 or Parkin exacerbates renal injury in UUO mice, supporting a protective role for mitophagy in CKD.

## Abnormal Mitochondrial Biogenesis

In response to changing energy demands triggered by developmental signals and environmental pressures, cells initiate mitochondrial biogenesis, the process of generating new mitochondrial mass and mtDNA replication through the proliferation of existing mitochondria (120). PGC1α is considered to be a master regulator of mitochondrial biogenesis, and its expression is upregulated upon increased energy demands (eg, fasting or exercise) or stress conditions (eg, cold or hypoxia) (121, 122). Mechanistically, PGC-1a is activated by phosphorylation or deacetylation, which then stimulates the expression of a series of nuclear transcription factors, including nuclear respiratory factor 1, 2 (NRF1/2), and subsequently, the initiation of NRF binding to ETC genes and mitochondrial transcription factor family (Tfam) genes, which are responsible for mtDNA transcription and translation (122).The current data acknowledge that mitochondrial biosynthesis results in an increase in OXPHOS capacity, a decrease in pathological oxidative stress and the repair of mitochondria-related dysfunction (Figure 3).

### Role in Cardiac Injury

Previous studies showed the essential role of mitochondrial biogenesis during adulthood, which involve conferring protection against long-term cardiac dysfunction, and in early embryonic cardiac development (123). Subepicardial biopsies from patients with HF exhibit decreased PGC1α expression (124). Consistent with the clinical finding, PGC1α ablation impairs mitochondrial energy metabolism and induces the development of HF in mice (125). Likewise, *in vitro*, dramatic reduction in mtDNA copy number in ischemic cardiomyocytes, which leads to reduced mitochondrial mass and increased mitochondrial fragmentation, while PGC1α overexpression attenuates these abnormalities (126). These findings suggest that downregulation of PGC1α facilitates the pathogenesis and progression of HF. However, the results of other studies sound a different tone. Hu's study showed no significant changes in PGC-1α gene expression in myocardial tissue of patients with congestive HF (127). Addition, in a transgenic mouse model, myocardial-specific PGC1α overexpression resulted in excessive mitochondrial proliferation and disruption of sarcoplasmic reticulum structure in cardiomyocytes, leading to cardiac enlargement with reduced myocardial contractile function (128), which indicate the overexpression of PGC1α does not improve mitochondrial function (129).This may be explained by the fact that the outcome of PGC1α overexpression and its effects on HF are tightly related to the activity and the interaction of mitochondrial biogenesis with other intracellular events. Altogether, the role of PGC1α in HF remains controversial, while the role of PGC1α in different types of HF needs to be further investigated.

### Role in Kidney Injury

Mounting evidence supports a beneficial effect of mitochondrial biogenesis in kidney injury and repair after AKI. PGC1α was highly expressed in proximal tubules with abundant mitochondria (130). Decreased expression of PGC1α in the kidney was observed in both renal IRI- or cisplatin-induced AKI animal models and in renal biopsy samples from AKI patients compared with controls (65, 130). Further studies demonstrated that in mice models of septic AKI, the levels of PGC1α and downstream OXPHOS genes in the kidney were suppressed proportionally to the degree of kidney injury and were restored to normal levels during kidney recovery (131), suggesting a negative correlation between PGC1α expression in kidney and AKI severity. The pharmacological activation of PGC1α accelerated the recovery of renal function after IRI in mice supporting the mentioned findings. Notably, mouse podocyte-specific overexpression of PGC-1 resulted in altered mitochondrial properties, including formation of giant mitochondria, increased expression of ETC and mitochondrial fusion genes, and enhanced podocyte proliferation and dedifferentiation, leading to proteinuria and glomerulosclerosis (132).

## Evidence for the Participation of Mitochondrial Dysfunction in the Pathogenesis of CRS

**Figure 4:**
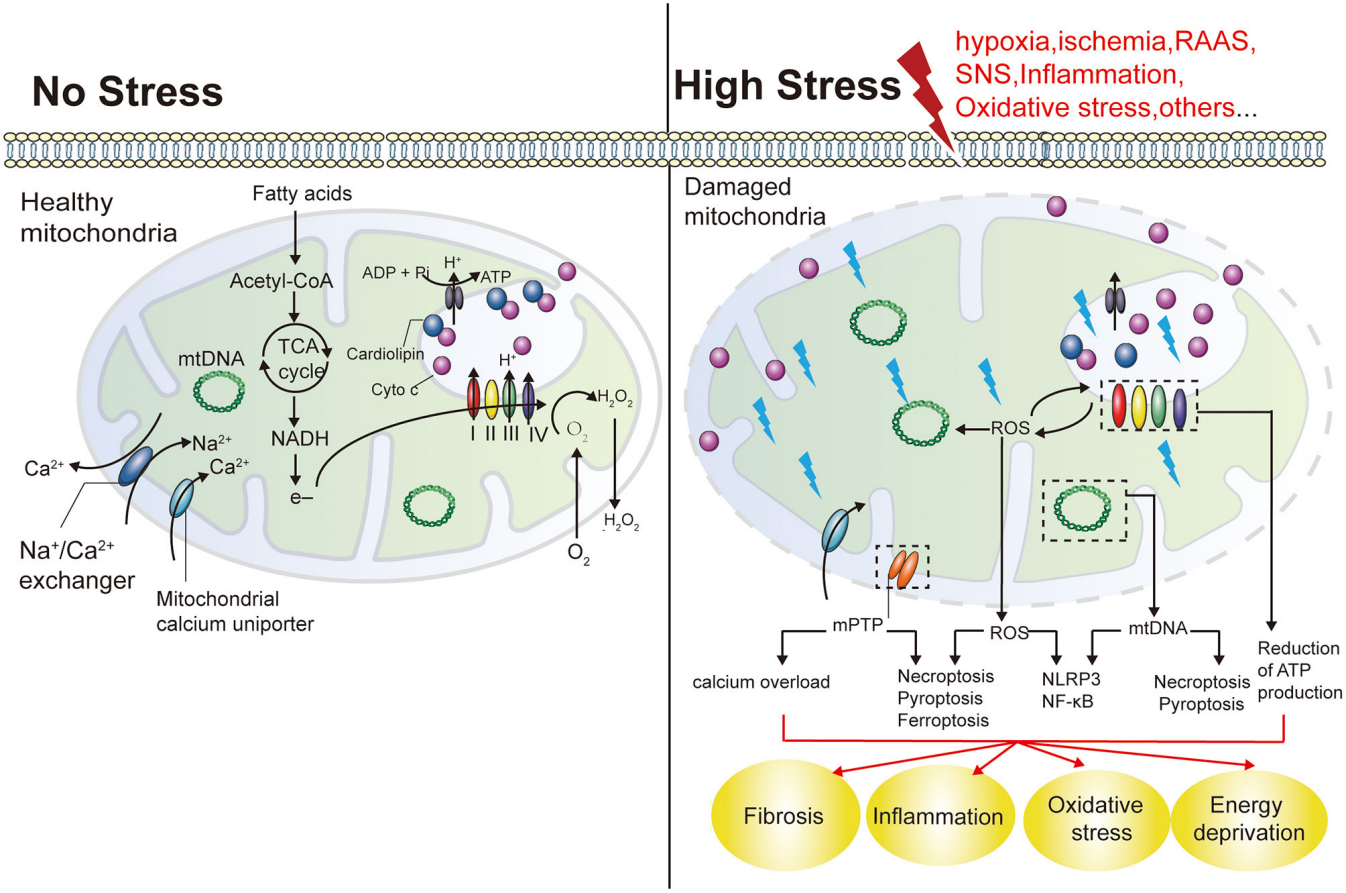
Mitochondrial functions and the effects of mitochondrial damage. Mitochondria play a key role in producing energy in the form of ATP. NADPH are formed by the oxidation of fatty acids and the cycle of TCA in the mitochondrial matrix, and their electrons are transferred to O_2_ through the electron transport chain (including COX I and IV). This results in the generation of a proton gradient across the IMM to produce ATP. Cyto C exists in free form in the IMS, or is anchored in the IMM by interaction with cardiolipin, acting as an electron carrier COX III and COX IV. Mitochondria are the main source of ROS. Mitochondria also play an important role in maintaining calcium balance in cells. Mitochondrial damage reduces ATP production and can result in the energetic failure of cells. An increase in mitochondrial ROS production by damaged mitochondria may also induce other forms of cell death, including necroptosis, pyroptosis and ferroptosis, as well as inflammation. NADH, nicotinamide adenine dinucleotides; TCA, tricarboxylic acids; IMM, mitochondrial intima; Cyto C, Cytochrome C; IMS, intermembrane space; IMM, intermembrane space; COX I, mitochondrial respiratory complex I; NLRs, nucleotide-binding and oligomerization domain-like receptors.

As previously mentioned, hemodynamic factors, RAAS, SNS, inflammation, and oxidative stress are core mechanisms of CRS, all of which synergize and activate each other, leading to further deterioration of cardiac and renal function (133, 134). In fact, all of the above factors cause mitochondrial damage in distal organs. First, mitochondria are the main consumers of oxygen in cells, and hypoxia directly or indirectly impairs mitochondrial dynamics, autophagy, and OXPHOS through the hypoxia-inducible factor pathway (56, 135). And one of the most deleterious effects of RAAS stimulation is the activation of NADPH oxidase, which results in increased mtROS in endothelial cells, renal tubular cells (136), and cardiomyocytes (137). Long-term SNS hyperactivity, on the one hand, promotes the growth of renal vascular wall and cultured cardiomyocytes through the production of mtROS (138, 139), on the other hand, it also promotes the activation of RAAS by directly stimulating the release of renin and plays a synergistic role in mitochondrial damage. In addition, as a hub of proinflammatory signaling, mitochondria are affected by elevated levels of multiple factors (such as C-reactive protein, IL-1β, IL-6, and TNF-a) in the chronic inflammatory environment of CRS (140, 141), further releasing inflammatory activators represented by mtDNA and ROS, triggering a vicious cycle of more severe inflammatory responses that predispose to fibrosis (142, 143) (Figure 4). Thus, mitochondrial dysfunction appears to play a key role in CRS. The role of mitochondrial dysfunction in different subtypes of CRS was summarized based on the available evidence (Table 1).

**Table 1:** Mechanisms and effects of mitochondrial dysfunction in different types of CRS.

**CRS types**	**Model**	**Mechanism(s) of action**	**Mitochondrial characteristics**	**Therapies**	**References**
CRS1	Asphyxia-induced cardiac arrest and CPR	Mitochondrial structure damage in renal tissue. ATP, SOD, CAT, CSH-Px, Opa1↓, Ca^2+^, MDA, Drp1↑	Suppressed mitochondrial fusion, enhanced fission	Levosimendan	(144)
CRS2	Modified approach of ACF	Swollen mitochondria and degenerated nuclei in RTCs, proapoptotic Bax, Cyt-c, c-casp3↑	Enhanced mitochondria-mediated apoptosis	—	(145)
	5/6 nephrectomy, DCM by DOX treatment	TGF-α, NF-κB, IL-1β, MMP-9, mitochondrial-Bax, c-casp3, cleaved-PARP, Smad3, γ-H2AX↑	Enhanced mitochondria-mediated inflammatory /oxidative stress/apoptosis	Empagliflozin	(146)
CRS3	Renal IRI	Myocardium ATP, ΔΨm, p-Akt, p-mTOR↓ ROS, fragmented mitochondria, IL-6, Grb2↑	Impairs cardiomyocyte mitochondrial bioenergetic	—	(23)
	Renal IRI	Fragmented mitochondria, Drp1, c-casp3↑	Enhanced mitochondrial fission and apoptosis	Mdivi-1	(147, 148)
	Renal IRI	Fragmented mitochondria in myocardium, ATP, ΔΨm, COXI, COXII, COXIV↓, p-Drp1, Mff, Fis1, Ca^2+^, MCU, IP3R, Cyt-c↑	Impairs cardiomyocyte mitochondrial bioenergetics and enhanced fission	Melatonin	(149)
CRS4	5/6 nephrectomy	Swollen-damaged cardiac mitochondria Cyt-B↓ Cyt-c, cleaved-PARP↑	Mitochondrial structure damage	Losartan (partially reversed)	(150)
	5/6 nephrectomy, DCM by DOX treatment	ΔΨm, PGC-1α, COXI, COXII, COXIV↓, ROS, p-Drp1, Mfn-2, Bax, c-casp3, LC3B1↑	Suppressed mitochondrial fusion, biogenesis and mitophagy	Entresto	(20)
	5/6 nephrectomy	Mitochondrial derangements, swelling, and vacuolation with disrupted cristae in cardiomyocytes, ATP, mtDNA, PGC-1α, ΔΨm, FAO, OXPHO↓, ROS, Glycolysis, Pit1, Pit2 IFR1↑	Suppressed mitochondrial biogenesis and OXPHOS	—	(22)
CRS5	Sepsis (intraperitoneal fibrin clots embedded with *S. aureus*)	mtDNA, NRF-1, NRF-2, TFAM↓, ROS, IL-6, IL-10, TNF↑	Suppressed mitochondrial biogenesis	—	(151)

*CPR, cardiopulmonary resuscitation; ATP, adenosine triphosphate; SOD, superoxide dismutase; CAT, catalase; CSH-Px, glutathione peroxidase; Opa1, mitochondrial optic atrophy 1; MDA, malondialdehyde; Drp1, dynamin-related protein 1; ACF, infrarenal aortocaval fistula; RTCs, renal tubular cells; Cyt-c, cytochrome C; c-casp3, caspase 3 activation; DCM, cardiomyopathy; DOX, doxorubicin TGF-α, tumor-necrosis factor-α; NF-κB, nuclear-factor-κB; IL-1ß, interleukin-1ß; MMP-9, matrix-metalloprotianse-9; PARP, poly-ADP-ribose polymerase; IRI, ischemia reperfusion injury; ΔΨm, mitochondrial membrane potential; COXI, mitochondrial respiratory complex I; COXII, mitochondrial respiratory complex II; COXIV, mitochondrial respiratory complex IV; Mff, mitochondrial fission factor; MCU, mitochondrial calcium uniporter; IP3R, Inositol 1,4,5-trisphosphate receptor; Mfn-2, Mitofusin-2; FAO, fatty acid oxidation; OXPHO, oxidative phosphorylation; IRF1, interferon regulatory factor 1; mtDNA, mitochondrial DNA; NRF, nuclear respiratory factor; TFAM, mitochondrial transcription factor A; TNF, tumor necrosis factor*.

### Mitochondrial Dysfunction in CRS1

CRS1 is defined as a sudden deterioration of cardiac function, leading to AKI and/or dysfunction (2). It usually follows acute ischemic or non-ischemic heart disease (152, 153), most commonly acute decompensated heart failure (ADHF) (154). Although the above mechanisms are risk factors for renal mitochondrial, there are limited studies on the direct relationship between CRS1 and mitochondrial dysfunction. Levosimendan is an effective drug for the clinical treatment of ADHF. A recent study showed that it decreased the risk of AKI after cardiopulmonary resuscitation in rats by enhancing mitochondrial respiratory enzyme activity, promoting mitochondrial energy metabolism, regulating mitochondrial dynamics-related protein expression, improving mitochondrial dysfunction, and reducing the number of apoptotic cells caused by mitochondrial pathways (144).

### Mitochondrial Dysfunction in CRS2

CRS2 is defined as chronic cardiac insufficiency, leading to progressive manifestations of kidney damage, which contributes to the progression of CKD (155, 156). Morphological studies in rat models of congestive HF-induced renal injury revealed mitochondrial swelling in renal tubular epithelial cells, possibly due to the release of Cyt-c, which mediated caspase 3 activation and nuclear transfer and triggered apoptosis, supporting the role of mitochondria-mediated apoptosis in CRS2 (145). Early empagliflozin treatment protects cardiac and renal function in CRS rats by reducing apoptin (mitochondrial-Bax/cleaved-caspase-3/cleaved-parp) and fibrosin (TGF-β/Smad3), reducing DNA/mitochondrial damage (γ-H2AX/cytoplasmic-Cyt-c), and maintaining mitochondrial function and integrity (146).

### Mitochondrial Dysfunction in CRS3

CRS3 refers to a CRS subtype in which the onset of AKI leads to the progression of acute cardiac injury or dysfunction (9). Proteomic analysis of CRS3 rats showed alterations in myocardial pyruvate metabolism, glyoxylate and dicarboxylic acid metabolism, starch and sucrose metabolism, and amino acid biosynthesis, with 23 proteins enriched in signaling pathways related to mitochondrial function, suggesting that AKI may affect cardiomyocyte metabolism or mitochondrial bioenergy (23). Growth factor receptor-binding protein 2 (Grb2) is a regulator of AKI-related myocardial injury, and Grb2 activation promotes mitochondrial metabolic disorders in cardiomyocytes by inhibiting the Akt/mTOR signaling pathway. Additionally, the administration of Grb2-specific inhibitors reverses myocardial pathological changes in the context of AKI (23). Furthermore, the dysregulation of mitochondrial dynamics caused by increased Drp1 expression and cardiac apoptosis plays an important role in AKI-induced myocardial injury (147, 148). Renal ischemia-reperfusion injury induces mitochondrial calcium overload in cardiomyocytes through the inositol 1,4,5-trisphosphate receptor (IP3R)-mitochondrial calcium uniporter (MCU) signaling pathway, decreasing mitochondrial membrane potential and increasing pathological mitochondrial fission. In addition, melatonin attenuates myocardial injury caused by cytoplasmic and mitochondrial calcium overload by inhibiting *IP3R* phosphorylation and *MCU* expression (23). Thus, Doi K et al. proposed that CRS3-related studies should center on the new concept of “mitochondrial hormesis” (149).

### Mitochondrial Dysfunction in CRS4

CRS4 is characterized by cardiovascular damage in patients with CKD in all stages (25). Pressure and fluid overload in CKD patients often lead to hypertrophy with histological changes, such as fibrosis (26). These changes are associated with inflammation and other cardiovascular factors, including hypertension, RAAS activation, or fluid overload, and are often accompanied by a decrease in GFR (157). Ang II is involved in CKD-induced myocardial interstitial fibrosis and cardiomyocyte hypertrophy by inducing mitochondrial structural damage and mitochondrial apoptosis. Ang II receptor blockers merely upregulate mitochondrial fusion-related protein levels, reduce mitochondrial swelling, and improve the spatial organization of cardiac mitochondria, suggesting that further identification of molecular pathways contributing to mitochondrial damage and appropriate intervention are essential in CRS4 (150). The drug Entresto protects cardiomyocytes and cardiac function in high-protein diet-fed CRS4 rats by regulating oxidative stress and *Mfn2*-mediated mitochondrial functional integrity (140). Hyperphosphatemia (HP) is a known serum hallmark of CKD. The transcription factor interferon regulatory factor 1 (IRF1) is a key molecule upregulated by HP through histone H3K9 acetylation, and it directly binds to the promoter region of HP-mediated *PGC1*α to play a role in its transcriptional repression. In contrast, the restoration of *PGC1*α expression or gene knockdown of *IRF1* significantly attenuates HP-induced changes *in vitro* and *in vivo*. These findings suggest that IRF1-PGC1α axis-mediated myocardial mitochondrial biosynthesis plays a crucial role in the pathogenesis of CRS4 (22).

### Mitochondrial Dysfunction in CRS5

CRS5 refers to systemic diseases (sepsis, drug toxicity, lupus, liver cirrhosis, or amyloidosis) resulting in simultaneous cardiac and renal damage and/or dysfunction (158). In sepsis-induced CRS5, the key pathway found to be altered is primary metabolism, which may affect local cycling through decreased ATP levels and mitochondrial dysfunction (151, 159). Tran et al. demonstrated that mitochondrial dysfunction, cell swelling, and marked acylglycerol accumulation in tubules led to reduced prostaglandin E_2_ and promoted medullary vasoconstriction in ischemic AKI (160). There is a clear correlation between the stage of mitochondrial dysfunction, disease severity, and prognosis of patients with septic myocardial injury (161). However, no studies have directly assessed cardiac and renal mitochondrial function in patients with sepsis.

## Concluding Remarks and Perspectives

This article reviewed the pathophysiological mechanisms of CRS from the perspective of mitochondrial dysfunction. The elucidation of the mechanisms by which two organs are interconnected is an unmet medical need. Given that mitochondria play a vital role in renal and cardiovascular disease (Figure 2), this organelle may be an excellent candidate therapeutic target to disrupt the vicious cycle between HF and AKI/CKD. Accordingly, we propose a three-step mechanism that may explain the pathophysiology of CRS. First, damaged renal tissue (heart) releases pro-inflammatory factors and oxidative metabolites into the circulation, and changes in the neuroendocrine system lead to the secretion of several hormones into the blood. Second, kidney (cardiac)-derived biomolecules interact directly with receptors or adaptors on the surface of cardiomyocytes (renal tubular epithelial cells) and have the potential to exert indirect effects on cardiomyocytes (renal tubular epithelial cells) through other mechanisms. Finally, as one of the most sensitive intracellular organelles, mitochondria sense a wide range of stimuli in the extracellular environment and respond to cardiac (renal)-derived biomolecules by changing their morphology. Impaired mitochondrial adaptation leads to insufficient ATP synthesis, which further triggers oxidative stress, inflammation, apoptosis, and fibrosis, possibly due to the transition from adaptive organ dysfunction to maladaptive organ dysfunction (Figure 2). However, this assumption has several limitations. First, receptors or adaptors expressed on the surface of cardiomyocytes and renal tubular epithelial cells have not been demonstrated. Second, mitochondria are not the only determinant of cytopathological changes. Cardiomyocyte injury can also be caused by mechanical stress due to intracellular acidosis, disturbed calcium metabolism, and fluid overload. Third, as discussed above, despite exciting preclinical data, translation of mitochondria-targeted agents into clinical use in HF and AKI /CKD remains a big challenge, for example, the results obtained in antioxidants in patients with heart or kidney diseases have been very diverse, and not all therapies as effective as preclinical studies have shown. Therefore, further studies on the relationship between mitochondria and other intracellular stress responses are needed to understand the sequence of events contributing to organ damage.

Given the key role of mitochondrial dysfunction in CRS, specific interventions targeting mitochondrial homeostasis to prevent and treat CRS have emerged as promising therapeutic strategies. A variety of compounds targeting mitochondria have been shown to prevent kidney injury and/or accelerate kidney repair in patients with AKI and CKD. Additionally, “mitotherapy” is considered a potential strategy for HF treatment and should help assess the use of these compounds in CRS. Emerging evidence suggests that mitochondria-targeted therapies acting upstream of cellular damage may have advantages over therapies targeting downstream processes (inflammation and fibrosis). Therefore, from this point of view, mitochondrial dysfunction may be one of the molecular links between cardiac and renal in CRS and is an emerging link in the pathophysiology of the diseases.

## Author Contributions

SS, BZ, and YL conceived and designed the study. XX and JL involved in database search and extracted the data. QJ, RC, and WX analyzed the data and wrote the manuscript. YL and YW polished the English. YW, HW, QS, and YH revised the manuscript. All authors listed approved it for publication.

## Funding

This work was supported by the Scientific and Technological Innovation Project of China Academy of Chinese Medical Sciences (Nos. CI2021A03323 and C12021A01603) and National Natural Science Foundation of China (Grant No. 81573799).

## Conflict of Interest

The authors declare that the research was conducted in the absence of any commercial or financial relationships that could be construed as a potential conflict of interest.

## Publisher's Note

All claims expressed in this article are solely those of the authors and do not necessarily represent those of their affiliated organizations, or those of the publisher, the editors and the reviewers. Any product that may be evaluated in this article, or claim that may be made by its manufacturer, is not guaranteed or endorsed by the publisher.
